# Zebrafish cornea formation and homeostasis reveal a slow maturation process, similarly to terrestrial vertebrates’ corneas

**DOI:** 10.3389/fphys.2022.906155

**Published:** 2022-11-01

**Authors:** Kaisa Ikkala, Sini Raatikainen, Henri Koivula, Frederic Michon

**Affiliations:** ^1^ Institute of Biotechnology, HiLIFE, University of Helsinki, Helsinki, Finland; ^2^ Zebrafish Unit, HiLIFE, University of Helsinki, Helsinki, Finland; ^3^ Institute for Neurosciences of Montpellier, University Montpellier, INSERM, Montpellier, France

**Keywords:** zebrafish, cornea, terminal differentiation, cell differentiation, maturation

## Abstract

Corneal blindness is the fourth leading cause of blindness worldwide. The superficial position of cornea on the eye makes this tissue prone to environmental aggressions, which can have a strong impact on sight. While most corneal pathology studies utilize terrestrial models, the knowledge on zebrafish cornea is too scarce to comprehend its strategy for the maintenance of a clear sight in aquatic environment. In this study, we deciphered the cellular and molecular events during corneal formation and maturation in zebrafish. After describing the morphological changes taking place from 3 days post fertilization (dpf) to adulthood, we analyzed cell proliferation. We showed that label retaining cells appear around 14 to 21dpf. Our cell proliferation study, combined to the study of *Pax6a* and *krtt1c19e* expression, demonstrate a long maturation process, ending after 45dpf. This maturation ends with a solid patterning of corneal innervation. Finally, we demonstrated that corneal wounding leads to an intense dedifferentiation, leading to the recapitulation of corneal formation and maturation, *via* a plasticity period. Altogether, our study deciphers the maturation steps of an aquatic cornea. These findings demonstrate the conservation of corneal formation, maturation and wound healing process in aquatic and terrestrial organisms, and they will enhance the use of zebrafish as model for corneal physiology studies.

## Introduction

The cornea is the transparent surface of the eye. Like skin, it serves as a barrier, protecting the eye from the external environment. In addition, the cornea is an important refractive structure of the light path and must therefore remain transparent if sight is to be preserved. The cornea contains three cellular compartments: the endothelium facing the frontal chamber, the collagen-rich stroma, and the multilayered epithelium on the outer surface. To retain transparency, the corneal epithelium renews constantly. This is done locally from the progenitor cells on the basal layer of the epithelium (Dhouailly et al., 2014), and globally from the stem cells located in the limbus, which is situated between cornea and conjunctiva (Schermer et al., 1986; Cotsarelis et al., 1989; Lehrer et al., 1998). The limbal stem cells give rise to the more centrally located progenitors, which renew the superficial cell layers. In the case of limbal stem-cell deficiency, among other conditions, corneal renewal is not maintained, leading to progressive blindness (Rama et al., 2010). Due its easy access, the cornea provides an excellent model for the study of epithelial cell dynamics in homeostasis and upon injury.

**FIGURE 1:**
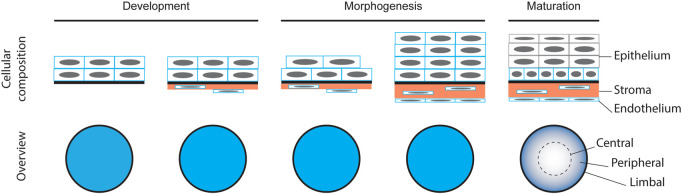
Schematic representation of corneal formation based on the existing literature.

Because of its conserved function, the formation of the cornea is similar across all camera-type eyed animals (Figure 1) (Miesfeld and Brown, 2019). The corneal epithelium differentiates from the surface ectoderm of the embryo after the lens has invaginated (Dhouailly et al., 2014). Simultaneous to the epithelial differentiation, cells of neural crest origin migrate below the presumptive corneal epithelium, giving rise to the corneal endothelial cell layer and the stromal keratocytes (Meyer and O'Rahilly, 1959; Pei and Rhodin, 1970; Johnston et al., 1979). As the cornea matures, the stroma thickens and the epithelium stratifies. While the human corneal epithelium stratifies from two to five layers upon the opening of the eyelid before birth (Sevel and Isaacs, 1988), in rodents this stratification occurs by 4 weeks of age (Chung et al., 1992; Song et al., 2003), and is likewise considered to be stimulated by eyelid opening (Zieske, 2004). The fully stratified corneal epithelium consists of the basal cell layer, the overlying superficial cells, and the terminally differentiated outermost cells.

Corneal growth, stratification and subsequent renewal require controlled proliferation. Studies of the corneal epithelial clonal pattern have indicated a switch from local to limbal renewal in three-to five-week-old mice (Collinson et al., 2002; Endo et al., 2007; Zhang et al., 2008), suggesting the establishment and activation of the limbal stem cell niche. Analysis of cell proliferation in central *versus* peripheral /limbal areas in 0- to 24-week-old mice showed that the limbal area contained the highest number of Ki67-positive cells, with the periphery containing the second highest number, and central cornea the lowest, throughout the stages studied (Kalha et al., 2018). Even in the embryonic stages E13.5 to E16.5, BrdU-labeling showed the highest proliferation rate in the peripheral regions, when samples were collected 1 h after BrdU administration to the pregnant female (Collinson et al., 2002). Collectively, these data suggest that the future limbal region is relatively active in cell proliferation well before the transition from local to limbal renewal, and that the central cornea retains a minimal proliferative activity in its mature state.

During the development and differentiation, the maturation status of epithelial cell populations is traditionally evaluated through the keratins that are expressed. The immature corneal epithelium is positive for *keratin 14* (*krt14*) (Richardson et al., 2017). The mature and terminally differentiated corneal epithelium is positive for *krt12*, co-expressed with *krt3* in human cornea, as well as rabbit and chicken cornea (Kasper et al., 1988; Chaloin-Dufau et al., 1990). The shift in *keratin* expression, from *krt14* to *krt12*, reflects the epithelium maturation process (Dhouailly et al., 2014). *Keratin 19* expression, on the other hand, was reported in the basal cells in limbal and peripheral regions in human cornea, and only limbal in mouse cornea (Yoshida et al., 2006). More recently, we showed that *krt8* and *krt19* expression in murine cornea changed from global expression perinatally to limbal expression only at 3 weeks after birth (Kalha et al., 2018). These patterns indicate the emergence of epithelial regions with specific differentiation status, the limbus and the periphery being less differentiated than the central region, and the superficial cells more differentiated than the basal cells.

The formation, growth and maturation of the cornea requires the proper expression of *Pax6* (paired box protein 6), which is one of the central transcription factors for ocular structure formation (Hill et al., 1991; Jordan et al., 1992; Quiring et al., 1994; Chow et al., 1999). In the corneal context, *Pax6* is expressed from early specification through development to adulthood (Koroma et al., 1997) and has been shown to promote the expression of the cornea-specific *krt12* (Liu et al., 1999). Silencing or knocking out *Pax6* in cultured corneal epithelial cells decreased the expression of *krt3* and *krt12* and increased the expression of skin-specific keratins *krt1* and *krt10* (Ouyang et al., 2014; Kitazawa et al., 2019), indicating that *Pax6* is necessary for the maintenance of the differentiated state in corneal epithelium. The loss of one *Pax6* allele, such as seen in the context of the rare disease called aniridia, characterized by an hypoplasia or lack of iris, as well as *Pax6* overexpression, showed overlapping effects such as reduced *krt12* expression, reduced adult corneal diameter or thickness, and altered wound healing (Ramaesh et al., 2003; Dora et al., 2008). Thus, an appropriate level of *Pax6* is necessary to maintain corneal integrity, as we have previously demonstrated in zebrafish cornea (Ikkala et al., 2022b).

During terrestrialization (Precambrian–Devonian), the corneal environment changed drastically, as the eye surface became exposed to a drier habitat. This change is reflected in the maturation process of the corneal epithelium (Zieske, 2004). In aquatic species such as zebrafish, the development, maturation, and growth of the cornea proceed while being immerged, and without the influence of the tear film. It is thus of great interest to compare corneal renewal in aquatic and terrestrial animals. The structural changes of the zebrafish cornea during development and maturation have previously been addressed (Soules and Link, 2005; Zhao et al., 2006; Akhtar et al., 2008). Pan and colleagues studied the clonal pattern of zebrafish cornea, and reported a shift from patchy pattern to limbal stripes in 4-week-old animals (Pan et al., 2013) This suggests maturation and adult renewal dynamics in zebrafish corneal epithelium similar to those observed in mice. Detailed studies on proliferation and molecular cues in the maturation process of the zebrafish cornea, however, are scarce.

Here, we describe zebrafish corneal epithelium maturation and growth with respect to corneal morphology, apical cell appearance, proliferation, and innervation pattern. In addition, we show the expression patterns of *pax6a* (paired box protein 6) (Krauss et al., 1991), and of *krtt1c19e*, a type 1 keratin previously reported as expressed in the basal epidermis of zebrafish (Lee et al., 2014). Together, these data depict the corneal epithelial maturation process. Finally, we describe the effect of an injury on corneal molecular markers, showing that a new maturation process occurs after wound closure, reflecting the loss of terminal differentiation status after a physical wound.

Collectively, our results demonstrate that zebrafish cornea behaves similarly to the one from terrestrial mammal when challenged. Furthermore, the recapitulation of developmental and maturation process after a physical wound could be of interest to understand pathological contexts, such as recurring abscesses. Finally, confirming zebrafish as a powerful model in corneal pathophysiological studies greatly benefits ophthalmological research.

## Results

### Corneal morphogenesis exhibits a long maturation process in zebrafish

Using detailed analyses derived from transmission electron microscopy, earlier studies showed that by 3dpf, all three main compartments of the cornea are present in the zebrafish: the endothelium, the stroma, and the epithelium (Soules and Link, 2005; Zhao et al., 2006). We investigated the overall morphology of zebrafish cornea during its morphogenesis, from 3dpf to adult stage, with histological sections.

**FIGURE 2:**
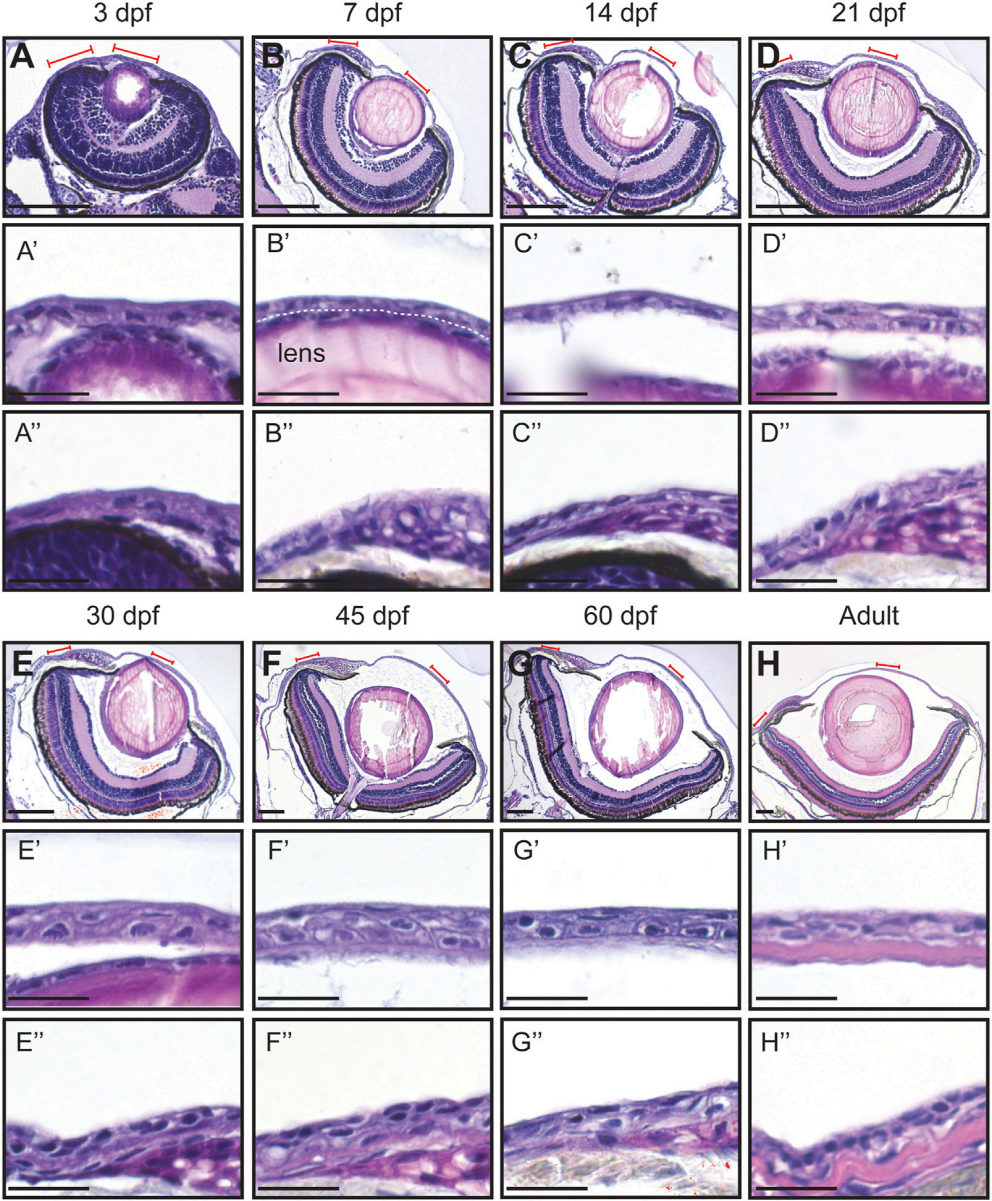
Hematoxylin-eosin staining of the eye in fish of different age (dpf, days post-fertilization). **(A–H)** Coronal sections of the eye, anterior side on the right. Red lines indicate the regions presented in higher-magnification images. White dashed line in **(B′)** indicates the border between the lens and the cornea. **(A’–H’)** Cornea in the central region. **(A’’–H’’)** Cornea in the periphery/limbus. 3 samples per age group were checked. Scale bars: 100 µm in **(A–H)**, 20 µm in **(A’–H’’)**.

At 3dpf, the cornea exhibited a bilayered corneal epithelium. The cornea was near the forming lens (Figures 2A,A’). Furthermore, the peripheral area was continuous with the skin (Figure 2A’’). At 7dpf, the conjunctiva started to form. Concomitantly, the morphology of the limbal area changed, but the epithelial compartment remained like the previous stage (Figures 2B,B’’). At 14dpf, the most striking changes observed were the increase in volume of the eye anterior chamber (Figures 2C,C’), and the limbus displaying various cell morphologies (Figure 2C’’). From 21dpf to 60dpf, central and peripheral corneal domains began to show signs of maturation, such as epithelial stratification, and limbus organization (Figures 2D–G’’). Finally, it was not until the adult stage that the stroma thickened, and the epithelium displayed a matured morphology (Figures 2H,H’’).

**FIGURE 3:**
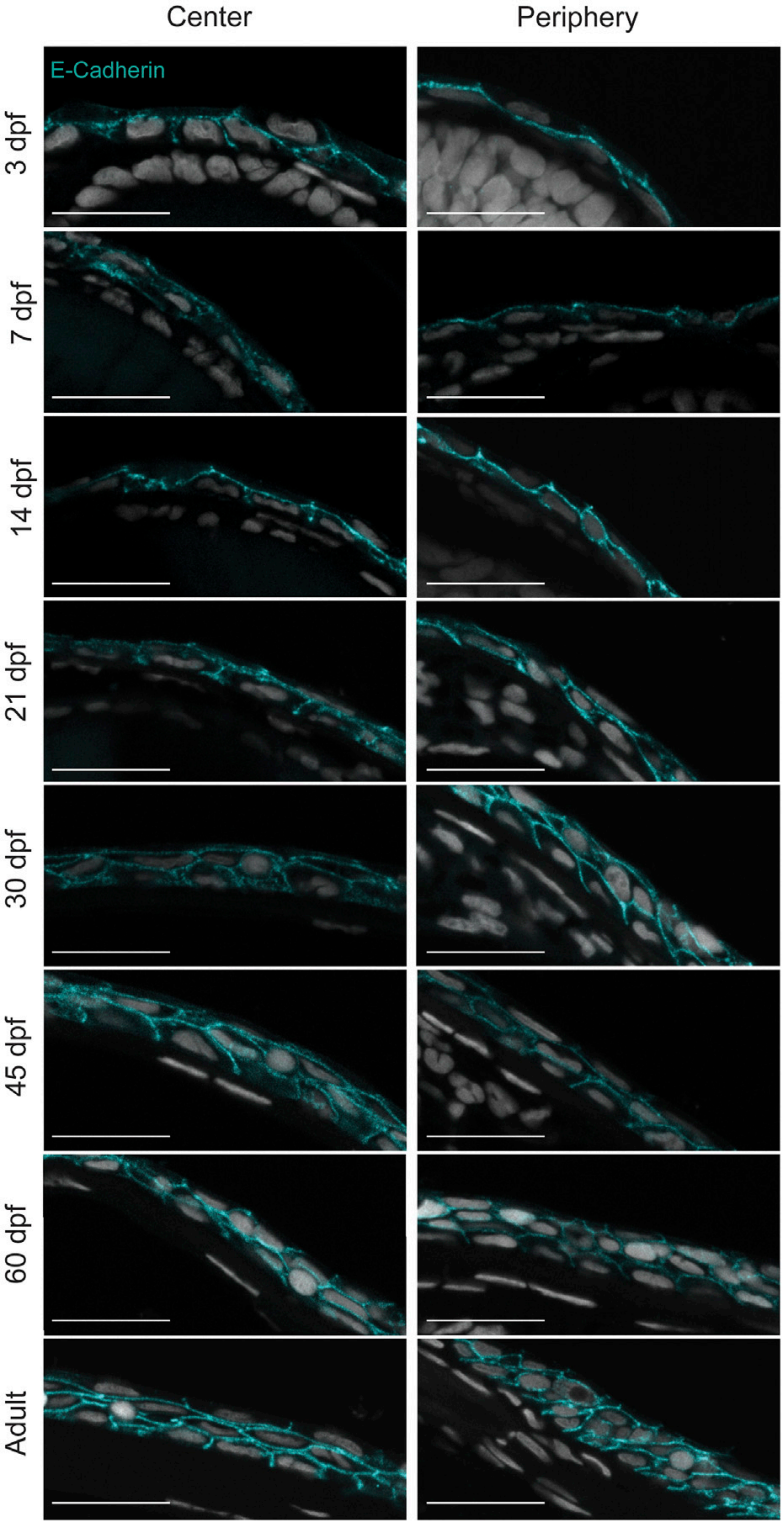
Stratification of the corneal epithelium. E-cadherin staining on formalin-fixed, paraffin-embedded sections showing the epithelial cell borders in different age groups. 3—5 samples were checked per age group. Scale bars: 20 µm.

The visualization of the epithelial cell morphology through E-Cadherin staining (Figure 3) confirmed our observations (Figure 3). For instance, the central corneal epithelium was bilayered from 3dpf, at 21dpf the epithelium was composed of 2-3 cell layers, and at 30dpf the epithelium had 3-4 cell layers; between the latter two stages its stratification became visible. Furthermore, the peripheral region showed a similar stratification pace. Finally, the adult cornea exhibited a multilayered central epithelium, organized as murine corneal epithelium, i.e. small basal cells covered by wing-cell like and wide superficial cells. Moreover, the peripheral domain contained morphologically different cell types. These observations indicate a slow maturation process in zebrafish cornea.

### Cell parameters confirmed the slow maturation process in zebrafish cornea

**FIGURE 4:**
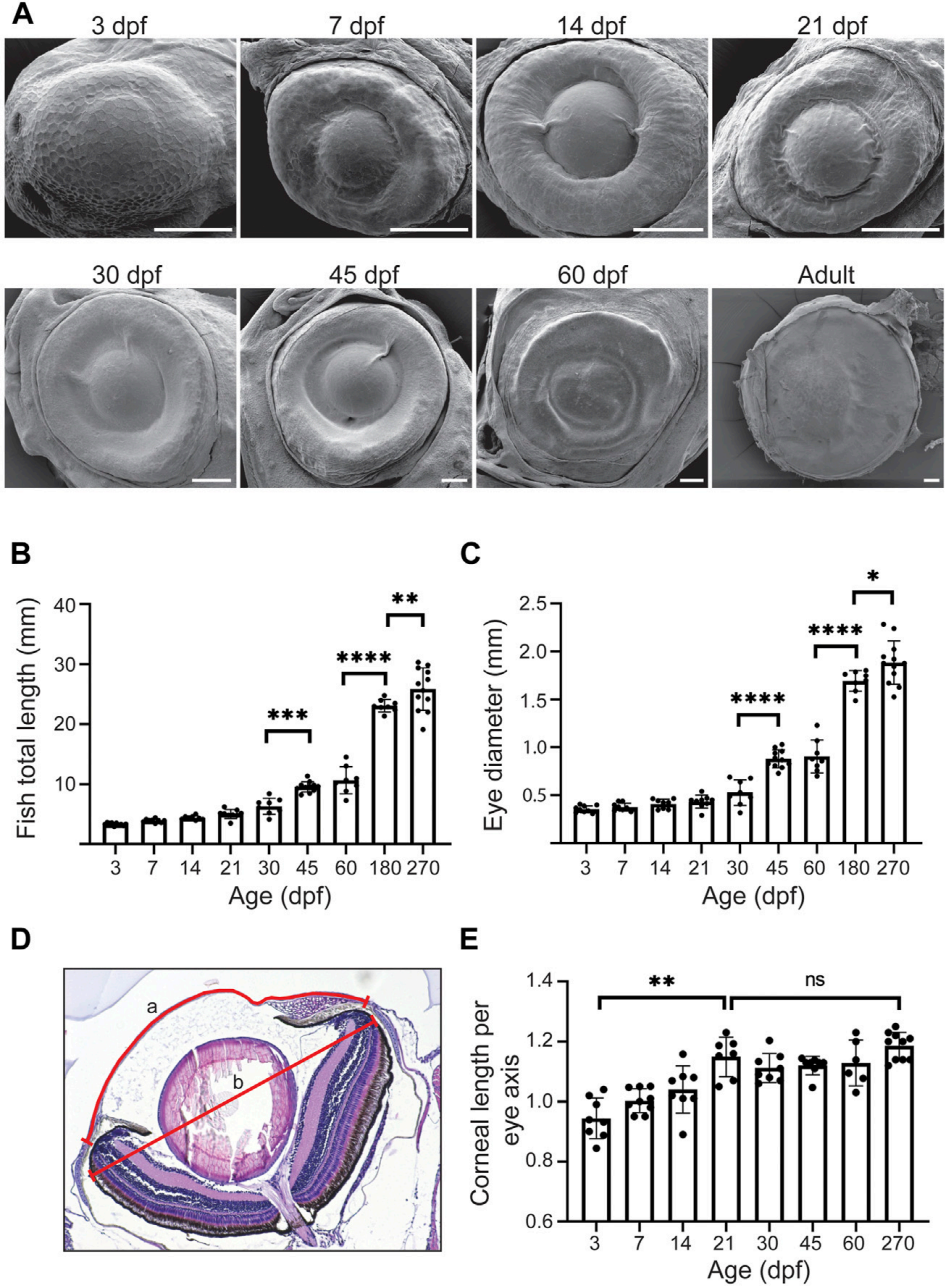
The growth dynamics of the eye and the cornea. **(A)** Representative SEM images of the eye in fish of different age (dpf, days post-fertilization, 3 samples were checked per age group). **(B,C)** Total length **(B)** and eye diameter on anterior-posterior axis **(C)** (n = 8–12, one-way ANOVA with Sidak’s multiple comparisons test). **(D,E)** Corneal length on anterior-posterior axis, normalized to the maximum eye diameter, shown in **(D)** and quantified in **(E)** (n = 6–10, Kruskal-Wallis test with Dunn’s multiple comparisons test). The results represent mean ± SD.

To get a sense of the corneal growth and maturation period, we visualized the eye surface with scanning electron microscopy (SEM) (Figure 4A). The overview of the eyes, together with histological data, suggested a drastic size increase from 30dpf onwards (Figure 4A). To gain a broader perspective on the corneal growth, we quantified the total fish length, eye diameter, and corneal diameter (Figures 4B–E). We observed modest, steady growth in the total length from 3 to 30dpf (from 3.3 ± 0.2 to 6.3 ± 1.3 mm), followed by swift growth between 30 and 45dpf (9.6 ± 0.9 mm). Finally, in the adult stage this growth continued between 180 and 270dpf (23.1 ± 1mm and 25.9 ± 3.5, respectively) (Figure 4B). The eye diameter followed a similar trend in growth (Figure 4C), increasing from 0.4 ± 0.0 to 0.5 ± 0.1 mm by 30dpf, then to 0.9 ± 0.1 mm at 45dpf (0.9 ± 0.2 mm at 60 dpf), and 1.7 ± 0.1 mm at adult stage. This would suggest that the cornea–covering the entire anterior eye surface in zebrafish - is likewise going through rapid growth between 30 and 45dpf, and still growing significantly from 60dpf to adulthood. Since the total corneal area is not solely defined by the eye diameter but also by the curvature of the corneal surface, we measured the corneal diameter along its anterior-posterior axis. When normalized to the axis of the eye, the corneal length increased significantly from 3 to 21dpf, and then this length stabilized (Figures 4D,E). This observation corresponds to the flat shape of the zebrafish cornea, especially as compared to human or mouse cornea. Hence, corneal growth in zebrafish is likely to follow the rate of growth of the eye, as the corneal length per eye axis is stabilized.

**FIGURE 5:**
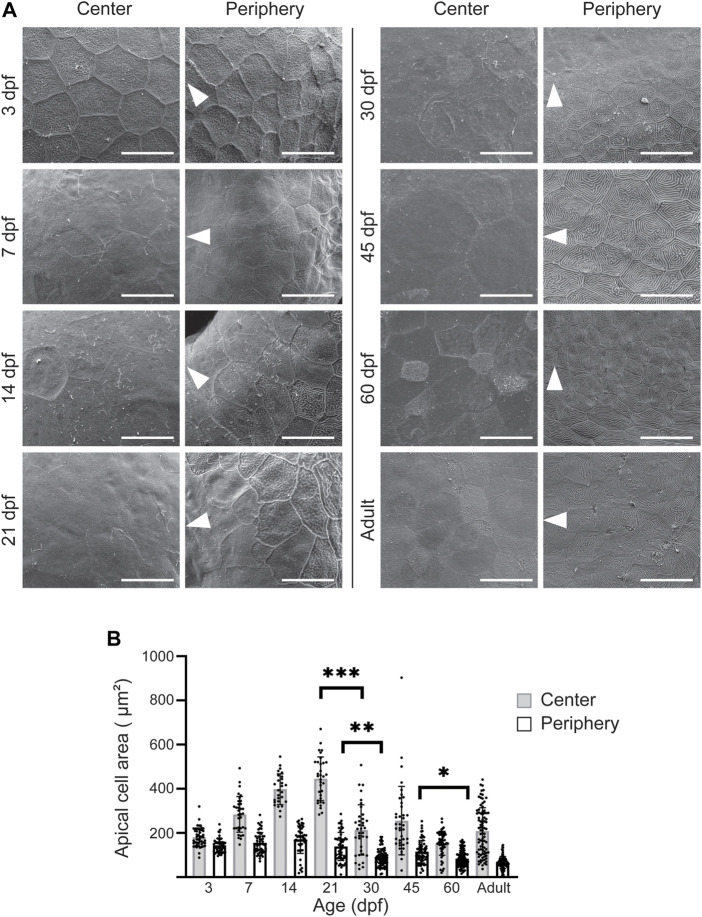
Apical cell appearance on zebrafish cornea. **(A)** Representative images of the central and peripheral regions. The white arrowheads point to the center of the eye. Scale bars: 20 µm. **(B)** Quantification of the apical cell area on central (gray) and peripheral (white) cornea. Cells from 3 eyes from 3 animals were pooled per age group for analysis (n = 27–87 in center, 47–121 in periphery, Kruskal-Wallis test with Dunn’s multiple comparisons test). The results represent mean ± SD.

Previously, we observed a clear distinction between the appearance of the central and the peripheral cell surface in adult zebrafish (Ikkala et al., 2022b). At the periphery, the apical cell area was remarkably smaller and the microridges more abundant, as compared to the central cornea. This difference could reflect the homeostatic renewal dynamics of the epithelium: on central cornea, the superficial cells are flatter, and lose the microridges as they desquamate off the eye surface. Here, we sought to determine the period at which this difference between corneal regions occurs. We studied age groups ranging from 3dpf to adulthood. At 3dpf, when the eye surface was still continuous with the surrounding skin (Figure 4A), the superficial cells had a polygonal shape with little size variation (Figure 5A). At 7pf, the cell-cell junctions and microridges became less evident, and the apical area of the central cells began to increase, as compared to the peripheral region. During the following 2 weeks, the peripheral cells became heterogeneous in their microridge appearance and size. Around 21dpf, we observed a rosette-like cell arrangement on the central cornea of some samples, as reported in a previous study (Pan et al., 2013), indicating cell protrusion (Supplementary Figure S1). From 30dpf onwards, the difference between the center and the periphery became more obvious, the central corneal cells being larger and more heterogeneous in size. Additionally, the peripheral cells showed abundant microridges.

We quantified the apical cell area in the SEM images (Figure 5B). Up to 21dpf, the superficial cells in the central corneal increased in average area, from 181.1 ± 42.1 to 445.7 ± 98.8µm^2^, followed by a sudden decrease to 214.3 ± 113.7 μm^2^ at 30dpf, and thereafter remaining below 260 µm^2^. For the same period, the peripheral region showed less pronounced changes, with an average apical cell area of more than 120µm^2^, and below 120 µm^2^ after 21dpf.

Taken together, the increase in corneal size and the decrease in the apical cell area suggest specific cell proliferation dynamics during corneal morphogenesis.

### A patterned proliferation fuels corneal growth and epithelial stratification

**FIGURE 6:**
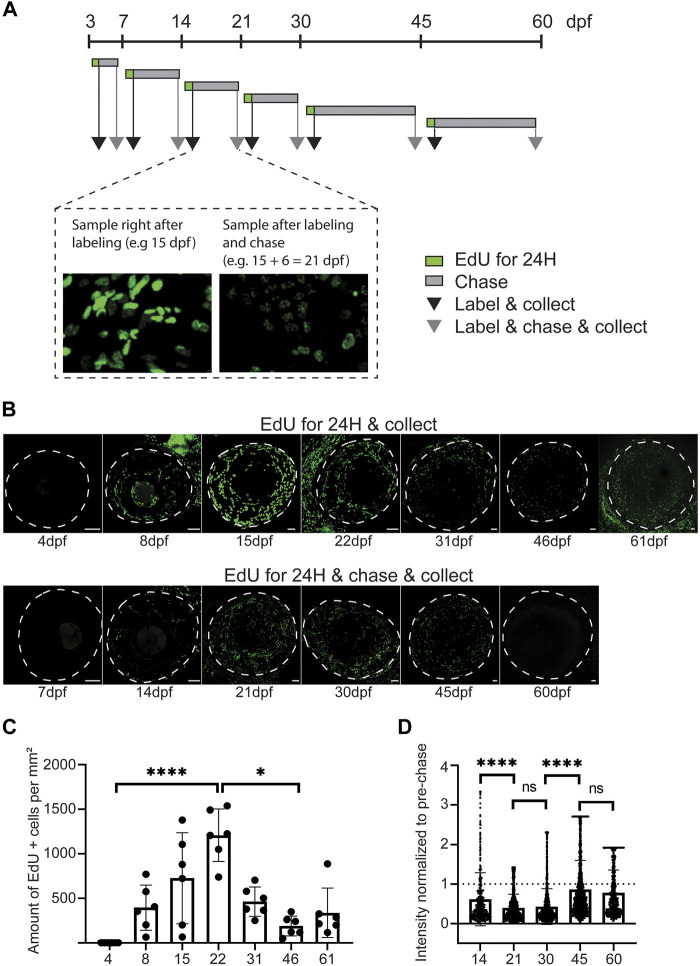
Proliferative activity on zebrafish cornea, as shown by EdU incorporation. **(A)** EdU was administered to the fish for 24 h at indicated ages. Samples were collected either right after labeling (black arrow), or after chase (grey arrow). **(B)** Representative images of whole mount samples on intact eyes. Dashed line indicates the eye border. Scale bars: 50 µm. **(C)** Quantifications of the proliferative activity from 3 to 61 dpf. **(C)** The EdU + cell amounts on the cornea right after labeling in samples collected at indicated ages. The bars show mean ± SD (n = 6, Kruskal-Wallis test with Dunn’s multiple comparisons test). **(D)** The mean signal intensity decrease of EdU + cells in samples collected after chase, relative to samples collected before chase. Each cell’s value was normalized to the mean pre-chase value of the labeling group in question. Cells from 3 eyes per group were pooled for analysis. The bars show mean ± SD (n = 383 for 14dpf, 1325 for 21dpf, 2,255 for 30dpf, 1682 for 45dpf, and 570 for 60dpf, Kruskal-Wallis test with Dunn’s multiple comparisons test).

After observing the changes in corneal diameter and apical cell appearance, we next investigated the cell proliferation pattern in zebrafish from 4 to 61dpf (Figure 6). The experimental process (Figure 6A) was as follows. We administered EdU to the fish for 24 h at each stage, and corneas were collected either right after labeling or after a chase. We quantified the labeled cells and measured the mean EdU signal intensity after chase (Supplementary Figure S2). With this specific experimental design, we discovered a drastic increase in cell proliferation from 3 to 21dpf (Figures 6B,C). This peak of proliferation was concomitant with epithelial stratification (Figure 3). While significantly lower, cell proliferation was maintained from 21 to 60dpf. As expected, the signal intensity observed after chase confirmed the previous results (Figure 6D). The loss of EdU signal intensity was drastic between 14 and 30dpf, when the proliferation was significant. This loss was significantly lower from 30 to 60dpf.

**FIGURE 7:**
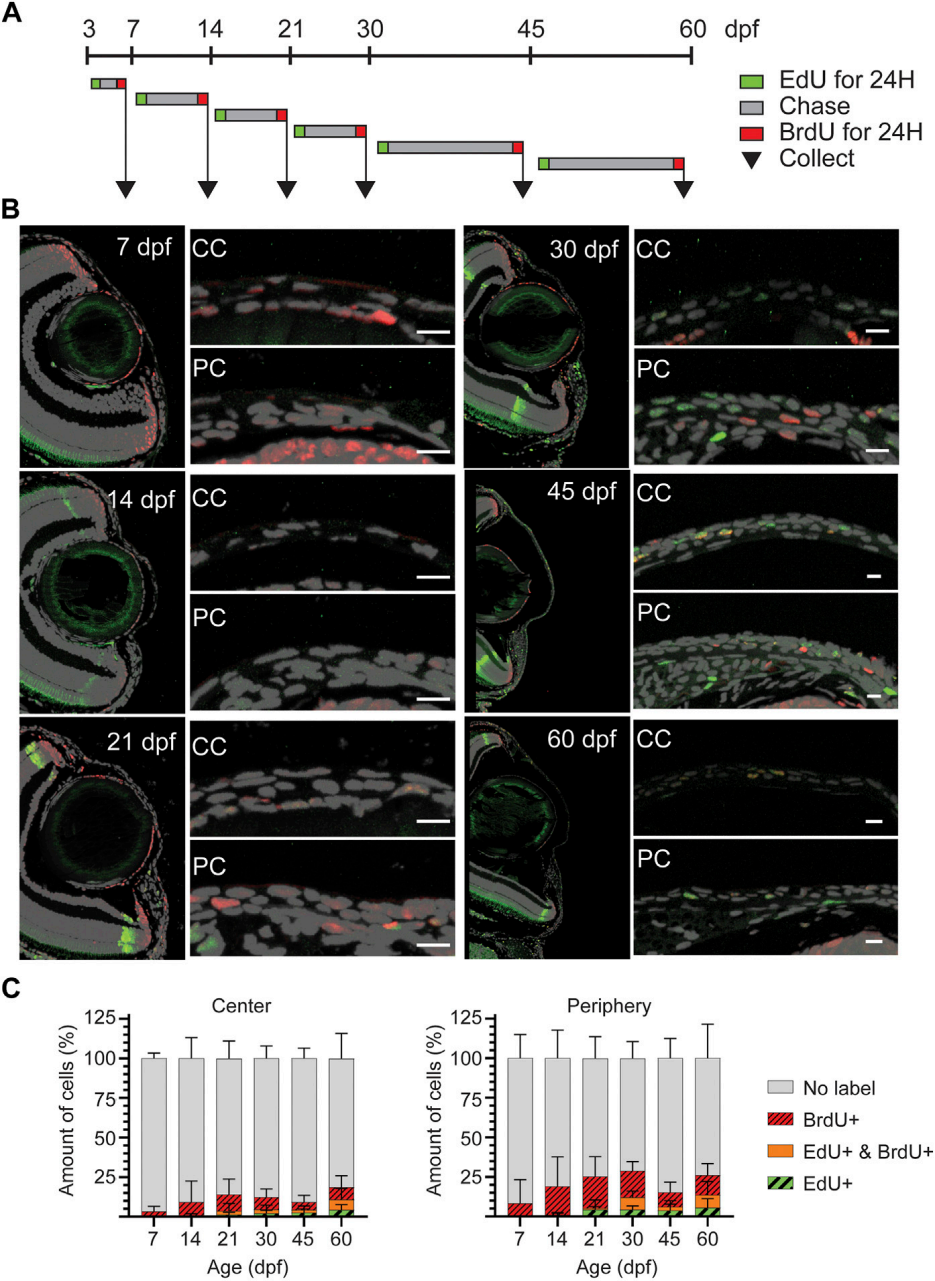
EdU/BrdU double-labeling on zebrafish cornea. **(A)** EdU (green) was administered to the fish for 24 h, followed by a chase period of 3–15 days. Then, BrdU (red) was administered for 24 h, and samples were collected. **(B)** EdU (green) and BrdU (red) staining on 5 µm paraffin sections. The panel shows the overview of the anterior eye, and central as well as peripheral/limbal regions of the cornea. **(C)** Quantification of EdU+, BrdU+, double positive, and EdU/BrdU-negative cells on the central region (left), and the peripheral region (right). Results represent mean ± SD (n = 6–9). The mean value of 2—3 sections from the middle of the eye were used per fish. Scale bars: 20 µm. CC, central regions; PC, peripheral/limbal region.

To discriminate the slow and fast proliferating cells, we performed a double labeling during the time between consecutive age groups up to 60dpf. EdU was administered at the beginning, and BrdU was given at the end of each period, allowing for an EdU chase (Figure 7A). Thus, the EdU signal would identify those cells which had retained detectable levels of label after chase, and BrdU signal would identify cells whose proliferation was ongoing at the time of sample collection. Due to poor staining outcome for BrdU on whole mount samples, we quantified the labeled cells on sections (Figure 7B). Prior 21dpf, EdU detection was negligible (Figure 7C), reflecting the dilution of EdU due to a high proliferative state in corneal epithelium during that period. From 30dpf onwards, proliferating and label retaining cells were found in peripheral and central cornea, reflecting the presence of different cell populations with different proliferation dynamics. Interestingly, the BrdU+ and/or EdU + cells were more abundant in the peripheral cornea, where the stem cell niche is established. To ensure that these observations were not biased by cell apoptosis, we investigated cell death during corneal morphogenesis and maturation, and discovered very few apoptotic cells in corneal epithelium from 3dpf to adult (Supplementary Figure S3).

### Corneal innervation formation

**FIGURE 8:**
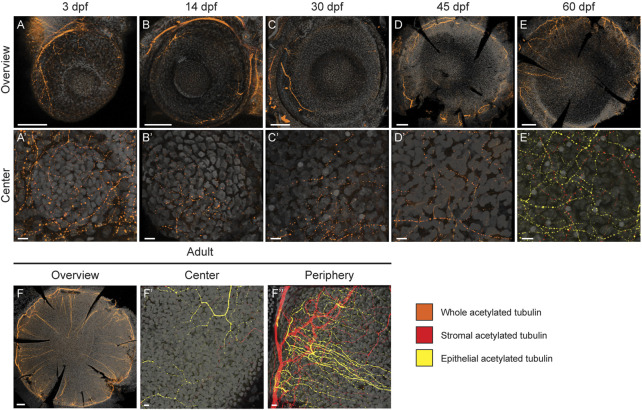
Corneal innervation in different age groups. Acetylated tubulin whole mount staining on maximum intensity projection images of whole eyes **(A–F)** and in central regions **(A′–F′)**. Additionally, the peripheral/limbal region is shown for adult stage **(F'')**. Orange color indicates the signal on the entire corneal tissue, yellow refers to the epithelial compartment, and red to the stromal compartment. During the first 1.5 months **(A′–D′)** the signal remains scarse on central cornea; the thickest neuronal branches can be observed in the peripheral regions **(A–D)**. At 60 dpf, the innervation is getting more dense, and distinct stromal (red) and epithelial (yellow) processes can be identified **(E,E′)**. In the adult cornea, thick peripheral nerve fibers branch and enter the epithelial compartment, forming an even, centrally-oriented pattern **(F–F)**. The epithelial *versus* stromal fibers organize to distinguishable networks **(F′,F)**. Three samples were checked per age group. Scale bars: 100 µm in the overview images, 10 µm in the magnified images.

Our results revealed a slow maturation process. It had been shown previously that the mature corneal epithelium receives coordinated signals to regulate its homeostasis. Among these signals, the neurotrophic factors from corneal innervation play an important role in epithelial renewal. While being heavily innervated, the innervation process and its final pattern differ between species (Lwigale, 2001; McKenna and Lwigale, 2011). Innervation plays a critical role in corneal physiology (Yang et al., 2018). The mammalian innervation consists mostly of sensory fibers (Muller et al., 2003), which appear gradually during corneal development and maturation (McKenna and Lwigale, 2011). In murine cornea, large nerve bundles are found in the stroma, with thinner neurites emerging from the stroma to innervate the epithelium. Here, we used Acetylated Tubulin antibody staining to detect an extensive part of the nerve fiber population, as previously reported in murine cornea (Bouheraoua et al., 2019). At 3dpf, we observed neuronal processes extending from the posterior peripheral region over the eye surface (Figures 8A,A’). Gradually, the thickest nerve trunks emerged around the peripheral cornea, giving rise to finer branches reaching towards the central region. Notably, from 14dpf to 45dpf, the central cornea showed far less signs of innervation (Figures 8B–D’). Only thin fibers were detected, and the innervation was sparce. However, at 60dpf we observed a denser network of nerve fibers, showing compartmentalization to epithelial (yellow) and stromal (red) fibers (Figures 8E,E’). In adult fish, this distinction between the epithelial (yellow) and stromal (red) innervation patterns was obvious (Figure 8F). Whereas the epithelial nerve branches were strongly orientated towards the center of the cornea, the stromal branches showed no specific orientation, forming a mesh. The most pronounced epithelial branches were localized at the basal epithelial cell layer, indicating that they would form the suprabasal nerve plexus in the zebrafish cornea. Finally, the adult stage was the only stage in which thicker neurites were observed in the central cornea, reflecting the late maturation of corneal innervation.

### Molecular maturation of corneal epithelium

While all our results demonstrate the late maturation of the zebrafish corneal epithelium, we chose two molecular markers to pinpoint the maturation time frame. First, the transcription factor Pax6 is expressed early on during eye development in presumptive ocular tissues of surface ectoderm and neuroectoderm origin (Nishina et al., 1999). In zebrafish, Pax6a protein was reported on presumptive corneal epithelium from 48hpf onwards (Macdonald and Wilson, 1997). Second, it was reported that the level of murine corneal maturation can be followed *via* the switch from *keratin 14* (*krt14*) to *krt12* expression in central cornea (Kao, 2020). Therefore, in addition to *Pax6a* expression, we investigated the expression of a type I keratin, *krtt1c19e*, which has previously been reported as present in the basal skin epithelium of zebrafish (Lee et al., 2014).

**FIGURE 9:**
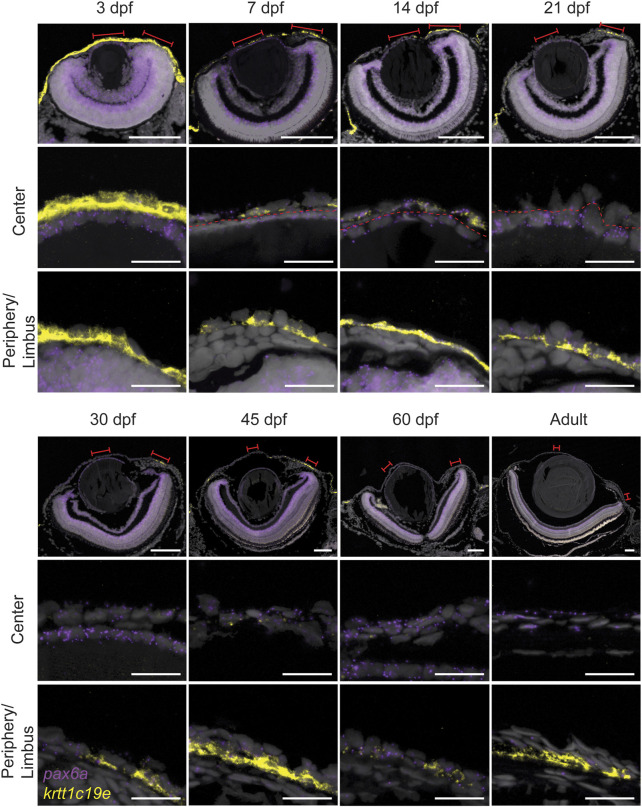
Expression of *pax6a* (red) and *krtt1c19e* (yellow) during corneal maturation. RNAScope *in situ* hybridization on 5- µm sections, red lines indicate areas in magnified images in center and periphery. Dashed line indicates the border between lens epithelium and cornea. 3 samples were checked per age group. Scale bars: 100 µm in the overview images, 20 µm in the magnified views.

Here, we used RNAScope to detect the *Pax6a* mRNA expression pattern during the corneal maturation process. At 3dpf, a low level of *Pax6a* expression was detected in the presumptive cornea (Figure 9). As the signal was masked by *krtt1c19e* expression, we decided to present a staining of *Pax6a* alone (Supplementary Figure S4). However, the signal observed, however, much lower than on the adjacent lens epithelium. From 7dpf to 21dpf, *Pax6a* expression was detected in most of corneal epithelial cells, at a low level (Figure 9). From 30dpf onwards, during the period of corneal stratification, the *Pax6a* signal increased, and was detectable in all corneal epithelial cell layers. Interestingly, krtt1c19e dynamic was inverted to the one of Pax6a. At 3dpf, we saw abundant expression throughout the surface of the embryo. From 7dpf onwards, the central cornea only showed a *krtt1c19e* signal occasionally, whereas the peripheral regions retained its expression in the basal epithelial layer, even in the adult stage. In general, the peripheral cell population positive for *krtt1c19e* became smaller during maturation and growth. Interestingly, in fish up to 45 days old, the posterior peripheral cornea, which houses most of the limbal stem cell niche, displayed a higher expression than the anterior peripheral region. These observations pointed to a corneal epithelial cell fate acquisition around 45 to 60dpf.

### Modulation of corneal epithelial markers during wound healing

**FIGURE 10:**
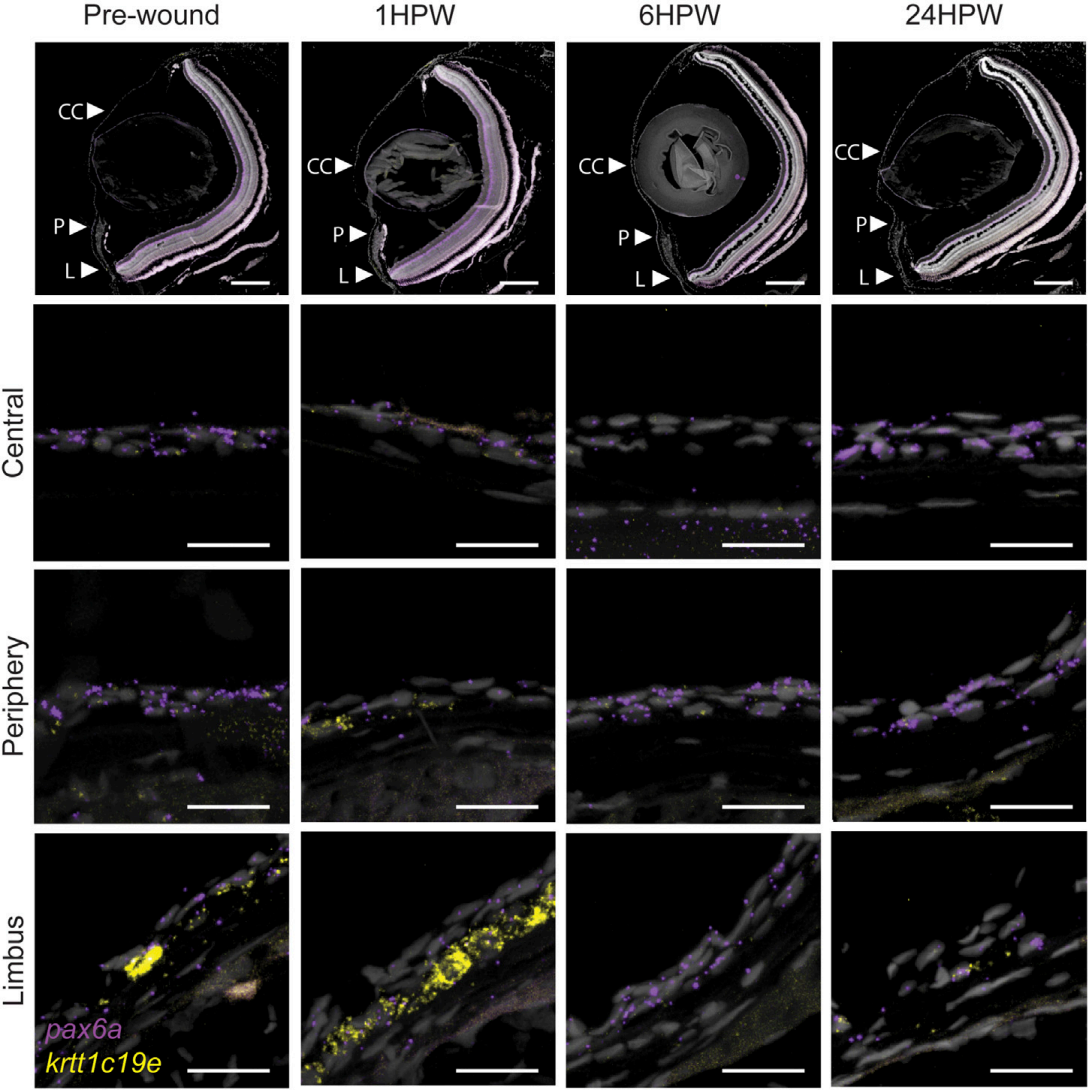
Gene expression changes in adult wound healing. RNAScope *in situ* hybridization on 5- µm sections showing the expression of *pax6a* (red) and *krtt1c19e* (yellow) before, and after 1, 6, or 24 h, an epithelial abrasion on central cornea. 3 samples were checked per age group. Scale bars: 200 µm in the overview images, 20 µm in the magnified views.

The dynamics of *krtt1c19e* and *Pax6a* expression during corneal maturation seemed to reflect the acquisition of specific cell fates in corneal epithelium. However, we have shown that Pax6a expression was suppressed during the wound healing process (Ikkala et al., 2022b). Therefore, here we followed the expression of these two markers during wound healing to investigate a possible reset of corneal maturation during that process. We created an epithelial surface wound mechanically with an ophthalmic burr, as previously described (Ikkala et al., 2022a). Before the wound, *Pax6a* expression was found in almost all epithelial cells throughout cornea (Figure 10). Furthermore, *krtt1c19e* was found predominantly in the limbus, and few cells were positive in peripheral and central cornea (Figure 10). Our findings confirmed results obtained previously (Ikkala et al., 2022b), namely the loss of *Pax6a* expression in the central cornea during the wound healing. The *krtt1c19e* expression exhibited an interesting dynamic: 1 h post-wound (1hpw), we detected an increase in its special of its expression, insofar as we detected its presence in the peripheral region, and a strong presence in the limbus. At 6hpw, after the wound closed and the restratification was ongoing, *krtt1c19e* expression was abruptly and totally lost. It restarted slowly in limbal cells at 24hpw, reflecting a return to a physiological situation. Our observations revealed a loss of cell fate during wound closure, followed by a reset of corneal maturation.

## Discussion

The corneas of camera-type eyes are structurally similar across the animal kingdom, despite the various habitats colonized by animals possessing such eyes. The main conserved feature is the cornea’s transparency. From early development to late in life, maintenance of this transparency is crucial to sight. It requires a constant epithelial renewal and a total lack of vascularization. Interestingly, the camera-type eye resulted from a convergent evolution in vertebrates and cephalopods (Serb and Eernisse, 2008). While the vertebrate cornea is a part of the eye, the cephalopod cornea is independent, and is often described as a specialized skin folding, which has an opening permitting the anterior chamber to be in contact with seawater (Hanke and Kelber, 2019). Moreover, the corneal adaptation to various habitats is reflected in specific structural traits. For instance, our histology sections performed on zebrafish showed a thin stromal compartment when compared to murine cornea (Kalha et al., 2018). Strikingly, the stromal compartment in fish swimming in deep-sea is thicker and more rigid than in mouse (Parravicini et al., 2012). Therefore, while the role of the cornea in sight has been conserved, the structure has been fine-tuned to adapt to various environmental conditions.

**FIGURE 11:**
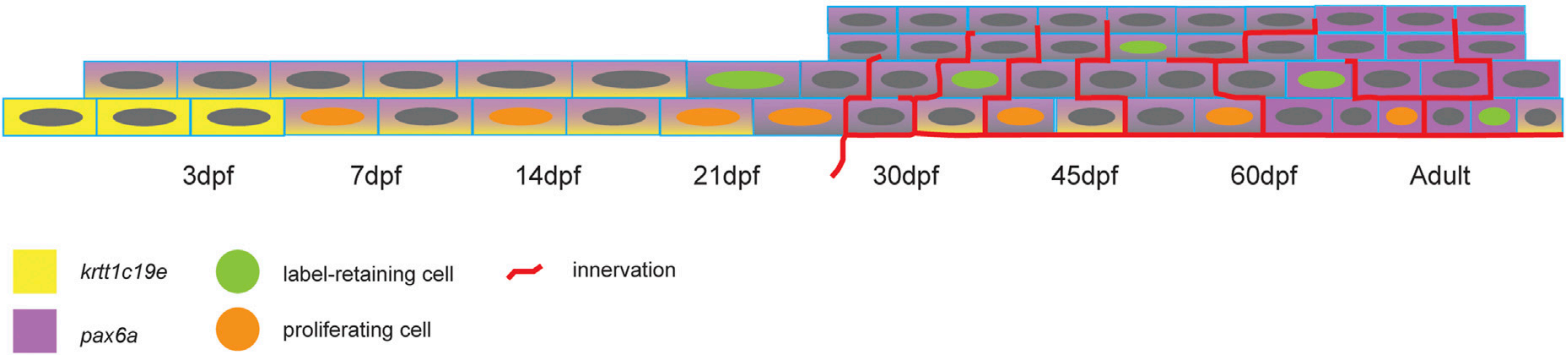
Scheme showing the change of *pax6a* and krtt1c19e expression, the appearance of proliferating and label-retaining cells, and the establishment of pronounced corneal innervation.

To date, the zebrafish corneal morphogenesis has been little documented. Our results show that despite an early establishment of the corneal tissue, the developmental and maturation processes are slow-paced. Our analyses of cell apical surface and proliferation dynamics demonstrated that the increase in corneal size is fueled primarily by cell size increase until 21dpf, when cell proliferation is at its peak. After that, cell apical surface shrinks and stratification occurs. This switch between cell enlargement and cell proliferation is important for stratification and cell fate acquisition. Indeed, it is at this time that the first label-retaining cells appear. This first period could therefore be called the corneal morphogenesis, from 3 to 21dpf (Figure 11).

Subsequent to this first period, corneal maturation begins. This maturation is one of the remarkable traits of the cornea. Numerous studies have reported the late corneal maturation in mice, beginning as the process of corneal stratification ends. The late expression of *krt12*, a marker of fully differentiated corneal epithelium, proves that the cornea is fully mature at about 3 months of age (Tanifuji-Terai et al., 2006). In this process, *krt14* expression, which is first present in all corneal epithelial cells, is progressively confined to the limbal area, where it marks the less differentiated corneal epithelial cells. Strikingly, *Pax6a* and *krtt1c19e* expression dynamics in zebrafish corneal epithelium can be compared to *krt12* and *krt14* dynamics in murine corneal epithelium. We observed that *Pax6a* expression progressively increased in corneal epithelium during the maturation process. However, the periphery /limbal territory displayed a very faint expression of this transcription factor. This observation corresponds to the expression dynamics of *krt12* in murine corneal epithelium. On the other hand, *krtt1c19e* expression in zebrafish cornea follows similar dynamics to those of *krt14* in mouse. By the end of the maturation process, we detected *krtt1c19e* only detected in the periphery/limbal domain. Our results show that the slow maturation is contemporary to epithelial stratification, a decrease of cell apical area, and the establishment of different cell populations, such as the label retaining cells, the *Pax6*+ cells in the central cornea, and the *krtt1c19e* + cells in the peripheral/limbal domain. Our data suggests that most of the maturation process happens between 21 and 45dpf, which is early in the zebrafish life.

Interestingly, both mouse and zebrafish can both live several years, about 3 and 5 years, respectively. Therefore, we face a vast discrepancy in the timescale of corneal ontology, as the cornea is mature at around 3 months of age for mouse and at 45dpf for zebrafish cornea. Our current hypothesis relates the timescale of corneal maturation to the lapse between birth and weaning. The postnatal and juvenile mouse is fed and protected, hence not relying much on sight, while the post-hatching zebrafish larva, at about 2dpf, relies on its own exploration and hunting, which requires functioning eyesight (Privat and Sumbre, 2020).

Another element of cornea which displays slow maturation is its innervation. In mouse, corneal innervation has been reported as fully mature by 4 months of age, which is a month after the completion of epithelium maturation (Bouheraoua et al., 2019). Our results showed a complete maturation of the corneal innervation in zebrafish between 2 and 9 months of age, which is later than the epithelium maturation. This feature of a delayed innervation maturation seems to be conserved. We hypothesize that it may reflect the necessity of molecular and mechanical cues from the epithelium to complete the tissue innervation. Those molecular cues could derive from guiding factors which are typically expressed after epithelium maturation, such as VEGF (Yu et al., 2008). The mechanical cues could be related to epithelial renewal, as occurs in murine cornea, the mechanical cues could be related to epithelial renewal, such as occurs in murine cornea; these cues could include the swirl seen in epithelial nerves (Bouheraoua et al., 2019) which mimicks the swirl seen in the epithelial renewal (Collinson et al., 2002).

Currently, the innervation patterning mechanism remains unknown. Strikingly, this pattern is not similar in all species. A recent report demonstrated that three main patterns exist in mammals, and that the patterns are different in mouse, rabbit, and pig (Marfurt et al., 2019). For instance, the rabbit corneal innervation pattern exhibits a horizontal patterning towards the inferonasal limbus, which is different to epithelial renewal (Haddad, 2000). Our data showed a centripetal innervation pattern, similar to the epithelial renewal dynamics (Pan et al., 2013). However, we did not detect swirl or vortex-like patterns in the central cornea. While we cannot exclude the possibility of failing to detect a subpopulation of corneal nerves with our antibody, this option seems unlikely as this antibody labels close to all corneal innervation in other species (Marfurt et al., 2019). We can therefore speculate that the guiding forces (molecular and/or mechanical) are comparable to those found in murine cornea, while they might differ at the very center of the tissue. Deciphering the cues responsible for corneal innervation mechanisms would produce invaluable new knowledge of corneal physiology and advance our understanding of corneal nerve pathologies such as neurotrophic keratitis.

Deciphering the corneal progressive morphogenesis and subsequent maturation is essential to understanding how this organ responds when challenged. Our previous work on murine corneal wound healing demonstrated the global transformation of corneal epithelial cell shape, and the mobilization of neighboring cells instead of limbal stem cells, with a lack of proliferation induction for the wound closure (Kalha et al., 2018). Furthermore, our recent work demonstrated a similar process during zebrafish corneal wound closure (Ikkala et al., 2022b). Our data presented here shows a drastic dedifferentiaton of corneal cells, as reflected in the increase of *krtt1c19e* expression, and the loss of *Pax6a* expression. Together with a change of cell morphology (Ikkala et al., 2022b), we speculate that the differentiated cells revert to developmental progenitors during the period of wound closure. This reversion returns the cornea back to the morphogenesis process, which is then followed by maturation. This high cell fate plasticity explains the recent reports which show that fully differentiated murine corneal epithelial cells can re-establish a limbal stem cell niche after its destruction (Nasser et al., 2018). The understanding of corneal formation brings a greater understanding of the corneal healing process.

Collectively, our results present the period 21-30dpf as crucial for zebrafish corneal maturation. We observed a decrease in cell apical area, the initiation of stratification, the peak of cell proliferation, and the establishment of label retaining cells, together with the start of molecular maturation. While none of these steps impacts on corneal transparency, given that the fish can see from early on, they are nonetheless crucial to the foundation of a physiological cornea. Remarkably, these events are similar to those which have been reported as following eyelid opening in murine cornea maturation, which occurs at 14 days after birth. This demonstrates the high degree to which corneal maturation and subsequent homeostasis has been preserved in both aquatic and terrestrial animals.

Taken as a whole, our results represent a much-needed advance in knowledge of zebrafish corneal biology. By deciphering the complete corneal ontogenic process and maturation timeline, this report provides the fundamental insight required to use zebrafish cornea as a study model for future research.

## Materials and methods

### Fish strains and maintenance

In this study we used the wildtype AB fish line, acquired from the Zebrafish facility (HiLife, University of Helsinki). The fish were kept in 14:10 light-dark cycle in standard conditions in the Zebrafish unit. For each stage studied, the fish were randomly chosen from the stock tank. Adult stages refer to fish aged 4–9 months old. The experiments were approved by the National Animal Experiment Board (lisence ESAVI/22167/2018 and ESAVI/1249/2022).

### Measurement of total fish length, eye diameter, and corneal growth

Fixed fish samples were placed on a plate, with millimeter paper underneath, and imaged. Images were opened in Fiji ImageJ 1.53. The scale was set using millimeter paper as reference, and total fish length and eye diameter (anterior posterior) were measured with the line tool.

We used histological sections for measuring the growth of corneal epithelium. Stained sections from the middle of the eye were imaged with AxioImager M2, and the images were opened in Fiji ImageJ 1.53. The width of the cornea was measured and normalized to maximum eye diameter (on the anterior-posterior axis).

### Scanning electron microscopy

Fish 3–45dpf (days post-fertilization) old were euthanized by prolonged anesthesia in 0.02% Tricaine solution (Sigma) on ice. 60dpf and adult fish were euthanized by anesthesia and decapitation. Fixing was carried out at +4°C in 2.5% glutaraldehyde (Grade 1, G7651, Sigma) in 0.1 M sodium-phosphate buffer pH 7.4. After approximately 24 h, samples were rinsed in 0.1 M sodium-phosphate buffer pH 7.4. Eyes were dissected and stored in 0.1 M sodium-phosphate buffer pH 7.4 at +4°C until further processing at the Electron Microscopy unit (University of Helsinki). Samples from fish 60dpf and older were treated with 2% osmium tetroxide (19,130, Electron Microscopy Sciences), dehydrated in increasing ethanol series and by critical point drying. For smaller samples from younger fish, the final drying was done by letting the ethanol evaporate at RT. Finally, the samples were coated with platinum. Images were taken with FEI Quanta Field Emission Gun scanning electron microscope.

### Apical cell size measurement

We quantified the apical cell area on central and peripheral regions of the eye surface using the scanning electron microscopy images (2,500X magnification) in Fiji ImageJ 1.53. The cell was manually outlined by the polygon tool, and the area was documented. Data were collected from three eyes per age group.

### EdU-labeling with or without chase, and analysis

We labelled proliferating cells on one 24-h time period per group, using 0.2 mM 5-ethynyl-2″-deoxyuridine, EdU (900,584, Sigma). Some fish were sacrificed, and samples collected right after labeling, the rest of the fish kept in regular maintenance until they reached the desired age. The material was processed into whole mount samples (as described below) and used for either counting the amount of EdU + cells in each stage (right after labeling), or for recording the mean EdU intensity change during the chase. In the quantification of the number of EdU-positive cells, we normalized the cell amount to tissue size. The size was recorded in Imaris, by manually defining the cornea in the image and creating a surface from Hoechst signal on the corneal area. The total area of the surface was used in the normalization of the EdU + cell amounts. For detecting the EdU signal intensity changes, the samples from the same labeling group (‘pre- and post-chase’) were processed together with similar staining and imaging conditions and compared to each other in the fluorescence intensity analysis. The intensity of the EdU + cells was defined by identifying EdU + nuclei in Imaris with the dots tool and recording the 488-channel intensity.

### EdU/BrdU-labeling, and analysis

We labelled proliferating cells on two 24-h time periods per group, using 0.2 mM 5-ethynyl-2″-deoxyuridine, EdU (900,584, Sigma), in time point 1 (e.g. at stage 14–15dpf), and 0.2 mM 5-bromo-2′-deoxyuridine, BrdU (B5002, Sigma) in time point 2 (at the following stage, e.g. 20–21dpf). The labeling reagent was diluted in E3 buffer or system-water, depending on the age of fish, from 10 mM (EdU) or 8–10 mM (BrdU) stock prepared in double-distilled water and stored at -20°C. The samples were processed into formalin-fixed, paraffin embedded sections and stained (as described below). For counting EdU+ and BrdU + cells, we imaged 2—3 sections per sample. Central cornea was defined as the region covering approximately 50% of the corneal horizontal length in the center of the tissue, the 25% on each side forming the peripheral regions. The dots function in Imaris was used to record the total amount of nuclei, and EdU+, the BrdU+ and double-positive nuclei, in the central and the peripheral regions.

### Corneal abrasion

The fish were anesthetized in 0.02% Tricaine and placed into an incision on a moist sponge, head protruding from the sponge surface. The eye surface was abraded with an ophthalmic burr (Algerbrush II, BR2-5, Alger Equipment Company) with a 0.5-mm tip, by pressing the rotating burr tip gently onto the eye and moving it with circular motion. Then the fish was placed back to the tank water.

### Whole mount staining

Fish were euthanized as above, and tissue fixed for 20 min on ice in 4% PFA (15,713, Electron Microscopy Sciences) in PBS. Then, samples were rinsed with PBS and stored in 100% methanol at -20°C. Upon staining, samples went through rehydration steps in decreasing methanol series (75/50/25% MetOH/PBS, 5 min each) at room temperature, and PBS rinsing. The eyes of adult fish were enucleated at this point.

For staining the corneal innervation, the samples were permeabilized in 0.5% Triton-X-100 (807,423, MP Biomedicals) in PBS for 1 h at room temperature, and blocked (10% goat serum (16,210,064, Life Technologies) + 0.5% BSA (A2153, Sigma), in 0.1% Triton/PBS) for 3—6 h at room temperature and incubated in primary antibody overnight at +4°C (1:200 in blocking solution). We used the primary antibody mouse-anti-acetylated tubulin (T7451, Sigma). Samples were then washed three times for 1 hour at room temperature in 0.1% Triton/PBS, blocked for 1—2 h at room temperature, and incubated in secondary antibody (goat-anti-mouse IgG, Alexa 568, A11057, Life Technologies) diluted 1:200 in blocking solution over night at 4°C. Finally, samples were washed as above, rinsed in PBS, counterstained in Hoechst (H3570, Invitrogen) 1:2000 in PBS for 30 min at room temperature, and mounted. For young stages, we mounted the tissue in 1% low-melting-point agarose (R0801, Life Technologies) on imaging dish. For fish ≥45dpf old, corneas were dissected and flat-mounted in 80% glycerol on microscopy slides.

In the EdU tracing on whole mount samples, the above protocol was used with the following modifications. The permeabilization was done in 0.3% Triton-X-100/PBS. For better tissue visualization in image processing, samples were stained with mouse-anti-E-cadherin (610,181, BD Biosciences, 1:200). After secondary antibody staining and washing on day 2, the samples were rinsed in PBS, and incubated 60 min at room temperature in the EdU detection reaction prepared according to kit instructions (C10337, Invitrogen), Subsequently, the samples were washed and counterstained with Hoechst as above. The lens was removed in fish older than 7/8 days, and the samples mounted in agarose for imaging.

### Stainings on formalin-fixed, paraffin-embedded sections

Fish were euthanized as above and fixed overnight in 4% PFA/PBS at +4°C. Samples were then embedded in Histogel (HG-4000, Thermo Fisher), and subjected to automated dehydration, xylene incubation, and paraffin embedding using the ASP 200 tissue processor machine (Leica Biosystems). Samples were sectioned with 5-m thickness on the coronal plane, dried overnight at +37°C, and baked briefly on a hotplate.

Upon EdU/BrdU staining, sections were rehydrated in decreasing ethanol series, permeabilized in 0.5% Triton/PBS for 10 min, and subjected to heat-induced antigen retrieval in automated retriever machine (62,700, Electron Microscopy Sciences) in 10 mM sodium-citrate buffer, pH 6.0. The sections were then washed in PBS for 10 min, blocked for 1 hour (10% goat serum in 0.1% Triton/PBS), and incubated with mouse anti-BrdU (RPN202, Cytiva) 1:100 in blocking solution overnight at room temperature. On the following day, the sections were washed in 0.1% Triton/PBS for 15 min, incubated in secondary antibody (goat-anti-mouse Alexa 568) 1:200 in blocking solution for 2 h at RT, washed in 0.1% Triton/PBS and PBS, both for 15 min. Sections were incubated in 3% BSA/PBS for 10 min. EdU tracing was done with the kit reaction components according to kit instructions (C10337, Invitrogen). Sections were then incubated again in 3% BSA/PBS for 10 min, washed in PBS for 10 min, incubated in Hoechst 1:2000/PBS for 20 min, washed in PBS for 10 min, and mounted in Fluoromount-G (0,100–01, Southern Biotech).

The TUNEL reaction for detecting apoptosis was done according to the manufacturer’s instructions (product 11684795910, Roche) in conjunction with E-cadherin staining. We used a similar immunofluorescence protocol as above, with mouse-anti-E-cadherin (610,181, BD Biosciences, 1:200) and donkey-anti-mouse 647 (A11004, Invitrogen). After the secondary antibody incubation step and washes, slides were again incubated in 0.1% Triton-X-100/PBS for 8 min at room temperature and rinsed twice in PBS. The TUNEL reaction mixture was prepared as per kit instructions, and further diluted 1:2 with PBS. The sections were incubated with the reaction mixture for 60 min at 37°C. A positive control samples were prepared by incubating the sections in DNAse I (50 U/ml, prepared in 50 mM Tris-HCl, pH 7.5, 1 mg/ml BSA) for 10 min at room temperature before TUNEL reaction. For a negative control sample, the label solution was used instead of the reaction mixture. Finally, the samples were rinsed twice in PBS, counterstained, and mounted as above.

Hematoxylin-eosin staining was done on rehydrated sections as follows. The slides were immersed in Hematoxylin (Harris’ Hematoxylin solution, Papanicolaou’s solution 1a, 109,253, Sigma) for 2 min, in running tap water for 3 min, and in eosin (Eosin Y, alcoholic, ht110132, Sigma) for 45 s. The sections were then dehydrated in increasing ethanol series, incubated in xylene, and embedded with mounting medium.

### RNAScope *in situ* hybridization

We detected the expression of *pax6a* and *krtt1c19e* with the RNAScope Fluorescent V2 kit (323,110, BioTechne), using probes Dr-pax6a 532,481) and Dr-krtt1c19e (1117231-C2), and Opal dyes 520 (1:1000 dilution) and 620 (1:1500 dilution) (FP1487001KT, FP1495001KT, Akoya Biosciences). We performed the staining on 5-μm paraffin-embedded, formalin-fixed sections, using manufacturer’s protocol with an additional baking of sections at 60°C for 30 min after deparaffinization. We performed a 17-min target retrieval, followed by a 20-min protease treatment.

### Light microscopy and image processing

The bright-field images on Figure 2 were obtained with Zeiss Axio Imager M2. The white balance and contrast were improved on whole images in Adobe Photoshop (Version 22.5.4). The images were re-scaled, cropped, and placed into figure panel in Adobe Illustrator (Version 25.4.1).

The fluorescently labeled samples were imaged with Leica SP8 inverted confocal microscope (Leica Microsystems, Wetzlar, Germany), by acquiring image stacks, and tile scans, when necessary, with HC PL APO 10x/0.40 CS2, HC PL APO 20x/0.75 CS2, or HC PL APO 63x/1.20 CS2 objective. The excitation/emission detection wavelengths were: 405/430–480 (Hoechst), 488/500–550 (Alexa 488, Opal 520), 561/570–650 (Alexa 568, Opal 620).

Signal intensity and channel colors were adjusted in Imaris software (Bitplane) or LAS software (Leica), and snapshots used for image panels. The epithelial and stromal compartments in Figure 8 were defined by the basal epithelial cell layer, which can be recognized by the even distribution and shape of the nuclei.

### Statistical analysis

Numerical data within a figure was tested for normal distribution. We used ordinary one-way ANOVA for normally distributed data, with Sidak’s multiple comparisons test. For data not normally distributed, or with small n-number, we used Kruskal-Wallis test with Dunn’s multiple comparison test. All statistical testing was done with Graphpad Prism (8.3.0). All groups within a figure were compared with each other, but only the most relevant test values are shown in each graph.

## Data Availability

The original contributions presented in the study are included in the article/Supplementary Material, further inquiries can be directed to the corresponding author.
