# Supplementary figures and images for: Zebrafish cornea formation and homeostasis reveal a slow maturation process, similarly to terrestrial vertebrates’ corneas
Source: Front Physiol. 2022 Nov 1;13:906155. doi: 10.3389/fphys.2022.906155 (PMC9663661; doi:10.3389/fphys.2022.906155)

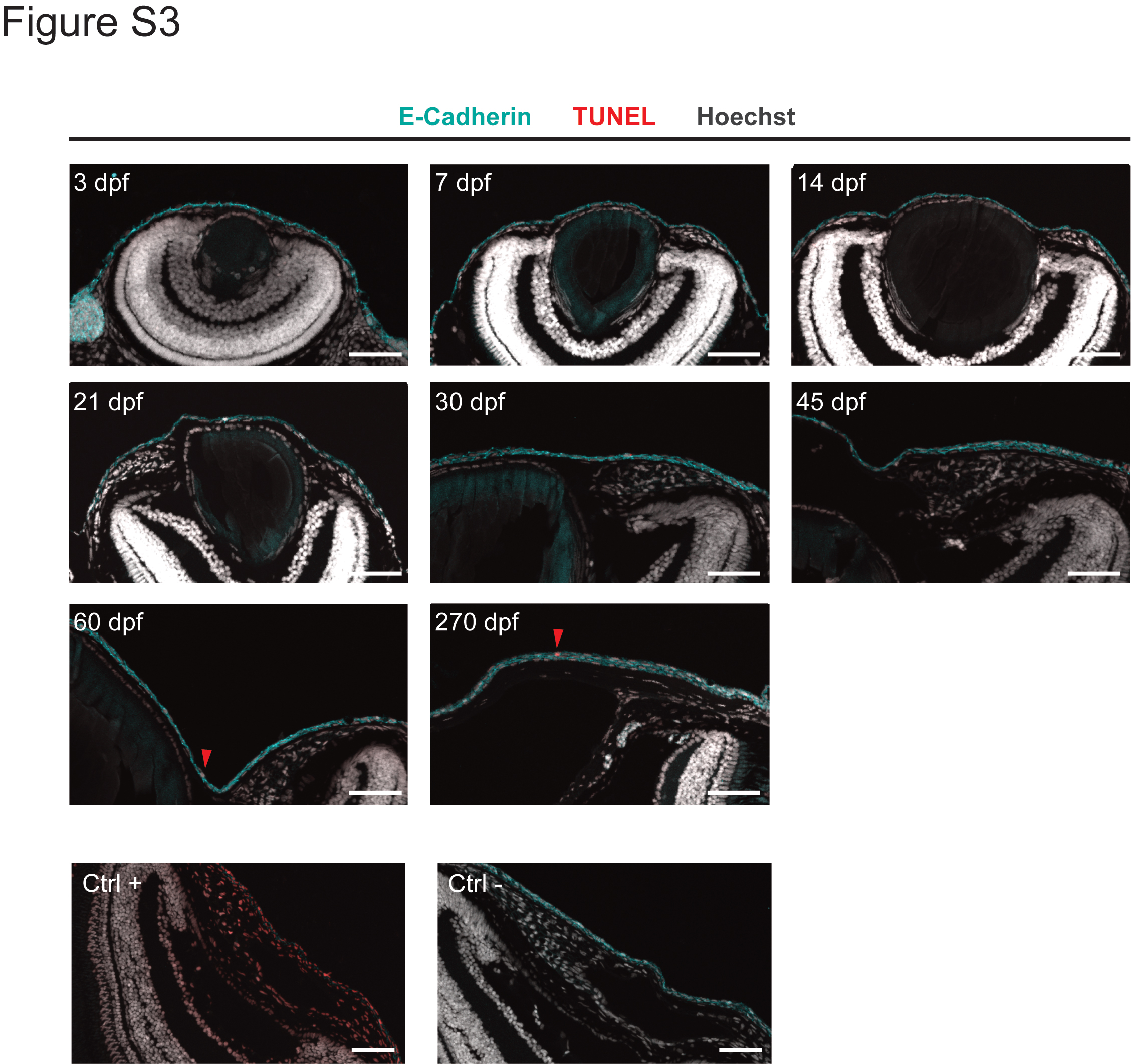

Supplement: Supplementary file 1 [file Image3.JPEG]

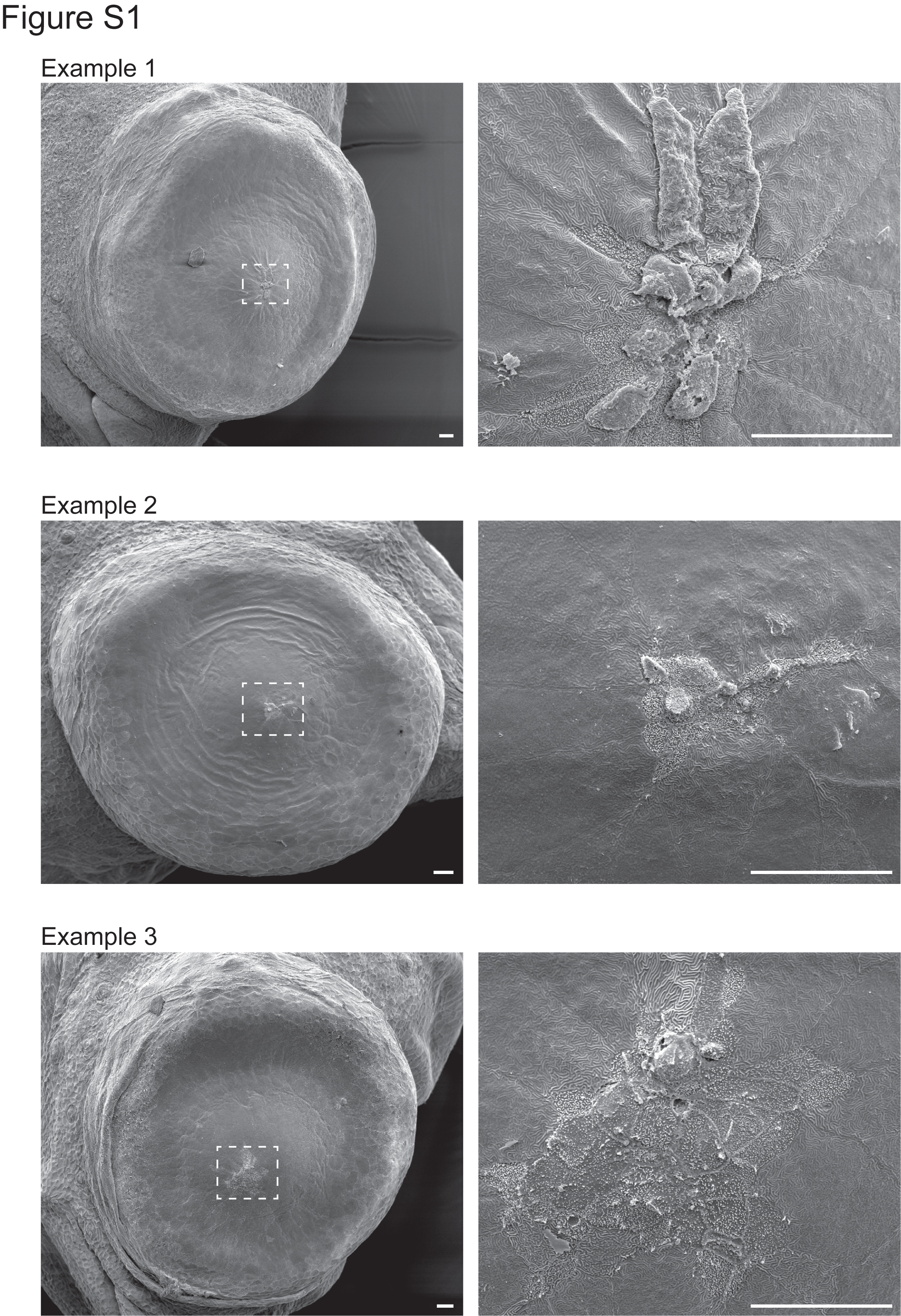

Supplement: Supplementary file 2 [file Image1.JPEG]

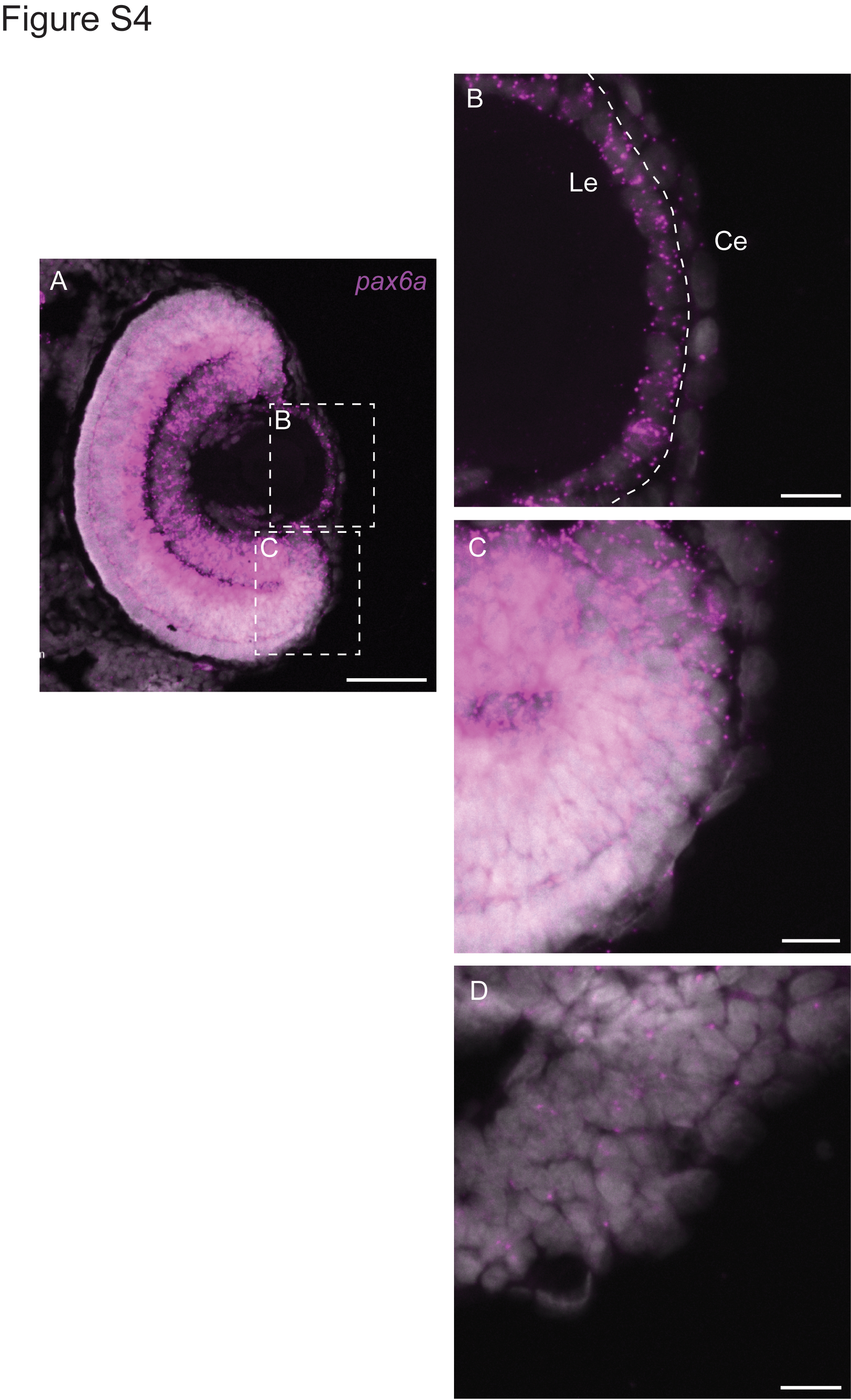

Supplement: Supplementary file 3 [file Image4.JPEG]

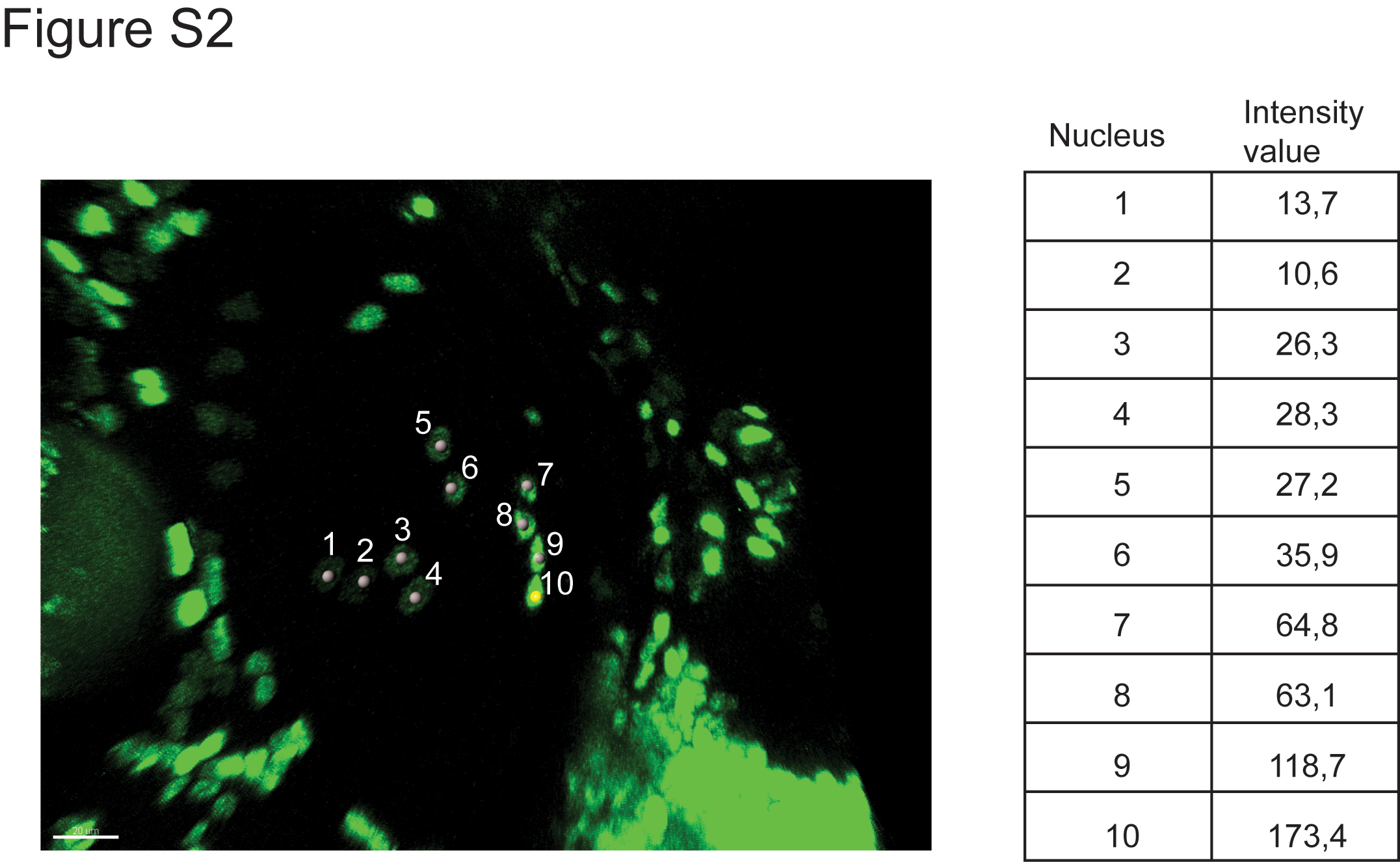

Supplement: Supplementary file 4 [file Image2.JPEG]
