# Supplementary material for: Zebrafish cornea formation and homeostasis reveal a slow maturation process, similarly to terrestrial vertebrates’ corneas
Source: Front Physiol. 2022 Nov 1;13:906155. doi: 10.3389/fphys.2022.906155 (PMC9663661; doi:10.3389/fphys.2022.906155)
Supplement: Supplementary file 5 [file DataSheet1.DOCX]

**Supplemental Figures**

**Figure S1.** Central cornea appearance in 21dpf fish. The dashed line points to the area presented in higher magnification. 5 samples were checked. Scale bars: 20 µm.

**Figure S2.** An example of the EdU signal intensity quantification. Values for 10 cells (annotated 1—10 on the image) are presented in the table.

**Figure S3.** TUNEL staining on formalin-fixed, paraffin-embedded sections from fish of different ages. The red arrowhead points to the rare positive cells. Three fish were checked per age group. Scale bars: 50 µm.

**Figure S4.** The *pax6a* signal on 3dpf cornea. A. Overview of a coronal section of the eye. B. Central region of the eye. Dashed line separates the lens and the cornea C. Peripheral region of the eye. D. Adjacent tissue. Le=lens epithelium, Ce=corneal epithelium. Scale bars: 50 µm in A, 10 µm in B-D.
